# Biological Selenite Reduction, Characterization and Bioactivities of Selenium Nanoparticles Biosynthesised by *Pediococcus acidilactici* DSM20284

**DOI:** 10.3390/molecules28093793

**Published:** 2023-04-28

**Authors:** Qingdong Wang, Chunyue Wang, Shanshan Kuang, Dezhen Wang, Yuhua Shi

**Affiliations:** 1 School of Life Sciences, Zhengzhou University, Zhengzhou 450001, China; qdwang@zzu.edu.cn (Q.W.); chunyuew@yeah.net (C.W.); sshankuang@163.com (S.K.); zzuwdz@163.com (D.W.); 2 Henan Key Laboratory of Bioactive Macromolecules, Zhengzhou 450001, China

**Keywords:** selenite reduction, biogenic selenium nanoparticles, *Pediococcus acidilactici*, antibacterial, antioxidant

## Abstract

Selenium (Se) is in great demand as a health supplement due to its superior reactivity and excellent bioavailability, despite selenium nanoparticles (SeNPs) having signs of minor toxicity. At present, the efficiency of preparing SeNPs using lactic acid bacteria is unsatisfactory. Therefore, a probiotic bacterial strain that is highly efficient at converting selenite to elemental selenium is needed. In our work, four selenite-reducing bacteria were isolated from soil samples. Strain LAB-Se2, identified as *Pediococcus acidilactici* DSM20284, had a reduction rate of up to 98% at ambient temperature. This strain could reduce 100 mg L^−1^ of selenite to elemental Se within 48 h at pH 4.5–6.0, a temperature of 30–40 °C, and a salinity of 1.0–6.5%. The produced SeNPs were purified, freeze-dried, and subsequently systematically characterised using FTIR, DSL, SEM-EDS, and TEM techniques. SEM-EDS analysis proved the presence of selenium as the foremost constituent of SeNPs. The strain was able to form spherical SeNPs, as determined by TEM. In addition, DLS analysis confirmed that SeNPs were negatively charged (−26.9 mV) with an average particle size of 239.6 nm. FTIR analysis of the SeNPs indicated proteins and polysaccharides as capping agents on the SeNPs. The SeNPs synthesised by *P. acidilactici* showed remarkable antibacterial activity against *E. coli*, *B. subtilis*, *S. aureus*, and *K. pneumoniae* with inhibition zones of 17.5 mm, 13.4 mm, 27.9 mm, and 16.2 mm, respectively; they also showed varied MIC values in the range of 15–120 μg mL^−1^. The DPPH, ABTS, and hydroxyl, and superoxide scavenging activities of the SeNPs were 70.3%, 72.8%, 95.2%, and 85.7%, respectively. The SeNPs synthesised by the probiotic *Lactococcus lactis* have the potential for safe use in biomedical and nutritional applications.

## 1. Introduction

Selenium (Se) is a crucial micronutrient for both humans and animals [1]. From a physiological perspective, its compounds have a broad range of biological functions such as its strong antioxidant capacity, immunity-enhancing effects, and its role in decreasing the risk of cancers, Keshan disease, Kaschin–Beck disease, cardiomyopathy, and so on [2,3]. Nevertheless, Se becomes an environmental hazard at high concentrations, and the uptake of more than 400 μg d^−1^ can cause serious health problems [4]. Selenium can be found in the environment in a variety of oxidation states (2, 0, +4, and +6) and in various forms such as unstable selenide (Se^2−^), water-soluble selenite (SeO_3_^2−^), selenate (SeO_4_^2−^), water-insoluble elemental selenium (Se^0^), and organic forms, such as selenocysteine and selenomethionine [3,5,6]. Due to their high solubility and bioavailability, selenite (SeO_3_^2−^) and selenate (SeO_4_^2−^) have the most biotoxic effects [7]. Selenium nanoparticles are characterised by low toxicity and their easy absorption compared with other forms of selenium [8]. In recent years, nano-selenium has been widely studied due to its unique features and functions [3,9,10]. Applications of SeNPs have been discovered in a number of areas, including the medical, therapeutic, biosensing, and environmental remediation fields [11]; therefore, it is of great significance to establish a green, effective and affordable biotechnology to convert poisonous selenite into innocuous elemental selenium nanoparticles.

At present, three main methods, are usually used to synthesise nano-selenium: physical methods, chemical synthesis, and biological technologies [5,12]. The physical and chemical synthesis processes are hazardous and difficult, and synthesised of nano-selenium has poor stability and is prone to agglomeration [13], so it is rarely used in medicine, feed, and as diet additives; however, studies have found that most microorganisms reduce or eliminate the toxicity of metal ions by changing the redox states of ions, and can thus be used to convert the more toxic inorganic selenium into SeNPs. Not only is the toxicity of inorganic selenium greatly reduced by these microorganisms, but it is also made easier to absorb and utilise, allowing its biological functions to be better exerted. Compared to the physical and chemical synthesis methods, the microbial method of SeNP synthesis has the advantages of being green, having a low cost, being highly efficient, and having an available source of raw material for production that is not limited by season [14]. More importantly, the surface layers of SeNPs prepared via microorganisms are generally coated with organic biomolecular layers, such as proteins and polysaccharides, which can enhance the stability of the SeNPs and effectively prevent aggregation [15]. Therefore, the synthesis of SeNPs by biological methods, especially via microbial transformation, has wide processing prospects.

Microorganisms have played a significant role in the human food chain as a result of the quick growth of fermented products. They participate in the creation of biomolecules and food fermentation, both of which are essential to maintaining human health. For humans, lactic acid bacteria (LAB) and many Lactobacillus genera are significant food-grade bacteria with a multitude of nutritional activities. Antibacterial activity, multivitamin production, and extracellular polysaccharide (EPS) synthesis have been demonstrated to be significant technical and functional properties of fermented food products. Calomme et al. (1995) were the first to propose that lactic acid bacteria can accumulate and transform selenium [16]. Through the direct intake of fermented foods made from selenium-rich lactic acid bacteria, the dual effects of nutrition and health care can be obtained while supplementing with selenium; moreover, the enrichment of selenium by lactic acid bacteria has the advantages of a short fermentation cycle and a simple process, as well as low toxicity and high efficiency. Hence, lactic acid bacteria are considered excellent carriers for selenium enrichment. In one study, *Lactobacillus casei* (*L. casei*) was found to produce many tiny selenium particles (lactomicroSel) (50–80 nm) [5]. Liu et al. found that when the concentration of sodium selenite was 37 μg mL^−1^ and cultured at 60 °C, the selenium-enrichment ability reached 43.46%, and there were no significant morphological changes in *E. durans* A8-1 cells [17]. Several recent studies have reported the beneficial effects of LAB on selenium-enriched fermented foods. In addition, Liu Dong et al. found that a yoghurt starter made with Se-rich strains not only had great fermentation performance, but also produced yoghurt containing a certain amount of organic selenium [18]. Similarly, Penas et al. (2012) also found that the biomass of lactic acid bacteria and the content of organic selenium in kimchi extract increased when sodium selenite was added to the natural fermentation process of white Brussels sprouts [19]. Therefore, the use of lactic acid bacteria to biosynthesise and transform inorganic selenium has become a research focus in the microbial production of Se-enriched products.

The objective of this study was to isolate a new efficient probiotic transformation strain with the potential to reduce sodium selenite to elemental selenium from alfalfa soils. The performances of the isolates were assayed under different growth conditions to evaluate their abilities to cope with the challenges of fermentation environments such as variable temperature, high salinity, and osmotic pressure. Selenium nanoparticles were synthesised in a green way based on lactic acid fermentation. Then, the bacterial cells were lysed using ultrasound, and the intracellular SeNP was isolated and purified using an organic-water extraction system. The synthesised SeNPs were characterised by transmission electron microscopy (TEM), dynamic light scattering (DLS), Fourier transform infrared (FTIR) spectroscopy, zeta potential analysis, and scanning electron microscopy (SEM). Their antimicrobial and antioxidant activities were evaluated.

## 2. Results and Discussion

### 2.1. Bacterial Isolation

There is a large number of published studies describing the role of selenium-rich microorganisms, such as *Bacillus subtillus*, *Providencia* sp. *DCX*, *Bacillus amyloliquefaciens*, *Proteus mirabilis*, *Lysinibacillus fusiformis*, and *Lactobacillus casei* [5,8,20,21,22]. At present, the research on selenium enrichment is very popular, and the elemental form of selenium is used as an additive in many foods, but there are few reports on enrichment of lactic acid bacteria with selenium. The aim of this study is to isolate a strain of Lactobacillus that can enrich selenium and provide a safe strategy for the production of selenium-rich functional food. In our work, a total of twelve strains producing red colonies were isolated from soil samples, and four different strains were identified by 16S rRNA sequence analysis. All strains were isolated from the same culture in MRS medium. LAB-Se2 and LAB-Se3 were of the same strain, as were LAB-Se1 and LAB-Se4. Similarly, isolates LAB-Se6, LAB-Se7, and LAB-Se8 were of the same strain. All isolates in the MRS medium with added selenite (100 mg L^−1^) produced red colonies on the agar plates (Supplementary Figure S1), as did the isolate in the selenite (100 mg L^−1^) liquid medium cultivation (Supplementary Figure S2).

### 2.2. Selenite Reduction and Se Nanoparticle Production by Four Isolated Bacterial Strains

Previous studies have assessed the bacterial growth of *L. bulgaricus* in the presence of Na_2_SeO_3_ at 10 mg L^−1^ and found that the bacterial growth rate was strongly reduced in the cultures containing 10 mg L^−1^ of Se [23]. However, this effect was not seen in our study, and it was found that Se did not inhibit the growth of the selected strains at the concentration of 100 mg L^−1^ sodium selenite compared with the control group without sodium selenite.

**Figure 1:**
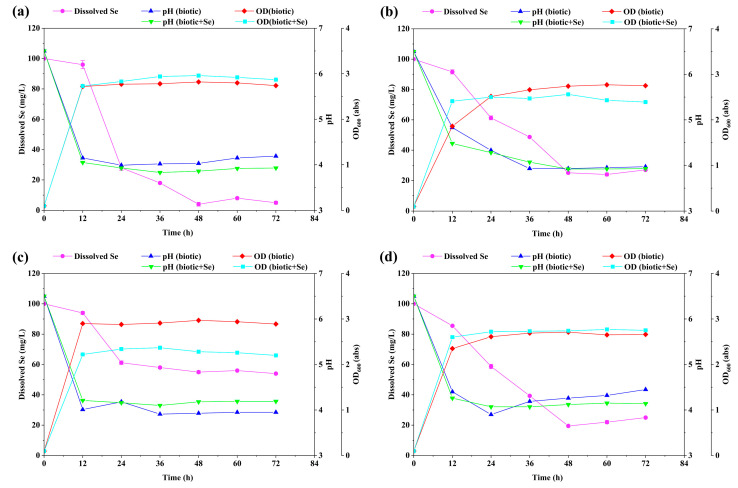
Bacterial growth and selenite reduction of strains (**a**) LAB-Se2, (**b**) LAB-Se4, (**c**) LAB-Se5, and (**d**) LAB-Se7, together with the biotic control without Se (IV) of each strain. Error bars indicate the standard deviation (*n* = 3).

During the growth of each bacterial strain, Se (IV) was reduced to elemental Se by a simultaneous decrease in Se (IV) concentration and a constant increase in bacterial cell density, which could be clearly measured by an increase in the optical density of the culture (Figure 1). All investigated strains showed a greater than 70% removal of Se (IV) at the end of the culturing period, except for strain LAB-Se5, which only reached 50% removal after 48 h of incubation. Changes in the OD, pH, and Se concentration observed in samples without the inoculation of the bacteria (i.e., in the negative control) showed that the selenite removal observed in all live cultures was catalysed by the microorganisms.

The optical density of strain LAB-Se2 increased to a maximum value (2.74 abs) after 48 h of culture. During the incubation of strain LAB-Se2 (Figure 1a), the cell density of the biotic control without Se (IV) was slightly lower than that with Se (IV), and the pH of the biotic control without Se (IV) was much higher than that with Se (IV). At 100 mg L^−1^ sodium selenite, the selenium removal was only at 4.8% after 12 h, and increased sharply to 72.3% within 24 h, finally reaching a saturation point of 98.1% at 48 h. Similarly, it was reported that *E. coli* ATCC 35218 showed 89.2% Se (1 mM) reduction power within 72 h of incubation in nutrient solution incubated at 37 °C [24].

Strain LAB-Se4 removed 80% of Se (IV) after 48 h of incubation (Figure 1b), and the pH of the culture increased to 3.86. The pH of the culture with Se (IV) was not significantly different from that without Se (IV). From previously published studies, the growth inhibition of *E. faecalis* strains CH121 and CH124 at 30 mg L^−1^ Se was 23% and 17%, respectively [25]. A similar result in this study was found in the growth of strain LAB-Se4, which was significantly inhibited at 100 mg L^−1^ of Se after 48 h of incubation. The OD values with and without selenium are 2.74 abs and 2.56 abs, respectively.

The capacity of strain LAB-Se5 to reduce selenite was only 54.2% at the end of a 48 h incubation period (Figure 1c), which was the lowest activity in this context. Hence, strain LAB-Se5 was not suitable for this study. The cell density of strain LAB-Se7 with or without Se (IV) was similar to the growth pattern of strain LAB-Se4 (Figure 1d). The cell density of both cultures had a slight reduction with and without Se (IV). The pH of culture media decreased from neutral to acidic. Therefore, strain LAB-Se2 was selected for further in-depth research.

### 2.3. Selenite Reduction and Se Nanoparticle Production by Strain LAB-Se2, at Various Initial pH Values, Temperatures, and Salinities

**Figure 2:**
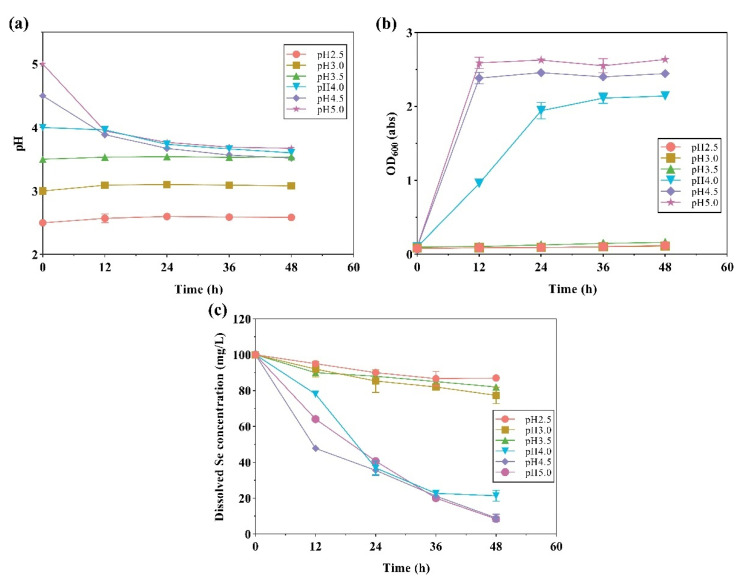
The effect of initial pH on pH changes, OD_600_, and selenite reduction by strain LAB-Se2. Changes in (**a**) pH, (**b**) optical density, and (**c**) dissolved Se (IV) concentration. Error bars indicate the standard deviation (*n* = 3).

Using our screened strain LAB-Se2, we first investigated the selenite removal ability of the strain under different pH conditions (Figure 2). In the initial screening process, we observed that the pH gradually decreased from neutral and eventually reached acidic, and the pH was basically stabilised at about 4.0 after 36 h of culture. Therefore, we assumed that the acidic environment may be ideal for strain LAB-Se2 to grow. The reduction of Se (IV) is a process that consumes hydrogen according to the half-reaction of Equation (1) [26], which leads to an increase in pH during the culturing.

SeO_3_^2−^ + 4e^−^ + 6H^+^→Se^0^ + 3H_2_O (1)

The results suggested that under the initial pH of 4.5 and 5.0, the removal rate of Se (IV) reached 90% after 48 h of culturing (Figure 2). Moreover, the pH value of the medium was stable at 3.5–4.0 (Figure 2a), which indicated that strain LAB-Se2 could produce acid to neutralise the base produced by Se (IV) reduction and adjust the medium to a pH value suitable for its growth (~4.0). This is similar to the reported growth of lactic acid bacteria, which secreted acidic substances to lower the PH of the medium [27]. Meanwhile, the growth of strain LAB-Se2 was significantly inhibited under strong acidic conditions (pH 2.5, 3.0, 3.5), and the growth was delayed at pH 4.0. After 36 h of culture, the OD value under pH 4.0 tended to be stable but lower than the OD value under pH 4.5 and pH 5.0 (Figure 2b), and the clearance rate of Se (IV) could still reach 78%. The growth delay of strain LAB-Se2 at pH 4.0 exhibited that the clearance rate of Se (IV) could be compensated by prolonging the growth time. In a previously reported study on the effect of selenium on the growth of strain NDSe-7, we found that there was also a growth delay at pH 10.0 [22]. The rapid growth of bacteria within 24 h was accompanied by a rapid decrease in the amount of Se (IV) (Figure 2c). It was speculated that the Se (IV) clearance rate below 90% at 48 h was probably because the growth of bacteria was limited at this time. This indicated that strain LAB-Se2 was more resistant to acidity above the appropriate pH.

**Figure 3:**
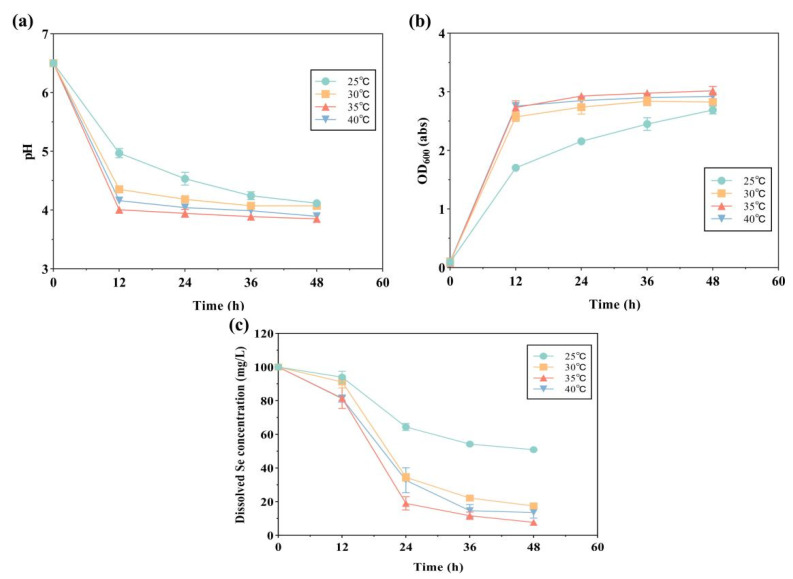
The effect of temperature on pH changes, OD_600_, and selenite reduction by strain LAB-Se2. Changes in (**a**) pH, (**b**) optical density, and (**c**) dissolved Se (IV) concentration. Error bars indicate the standard deviation (*n* = 3).

Like most bacteria, the growth of strain LAB-Se2 was gradually strengthened with the increasing ambient temperature (Figure 3). The Se (IV) clearance rate of strain LAB-Se2 was almost the same (more than 85%) during the 48 h incubation period at 30–40 °C, and the highest Se (IV) clearance rate was 92% at 35 °C. During the 48 h incubation period at 25 °C, the initial growth of strain LAB-Se2 was slow, but its OD value continued to increase and tended to the maximum value with the incubation time (Figure 3b). Its pH continued to decrease and tended to the lowest value (Figure 3a), but the Se (IV) clearance rate was still only about 50% at 48 h. Although the Se (IV) removal efficiency of strain LAB-Se2 was the highest at 35 °C, the Se (IV) removal efficiency at 30 °C and 40 °C was basically the same, and there was little difference from that at 35 °C (Figure 3c). In the range of 30 °C to 40 °C, the cell growth and pH trends were consistent but slightly different. Considering both the Se (IV) removal ability of strain LAB-Se2 and the temperature-dependent growth laws, a temperature of 35 °C was determined to be optimal for this strain to obtain the best performance.

**Figure 4:**
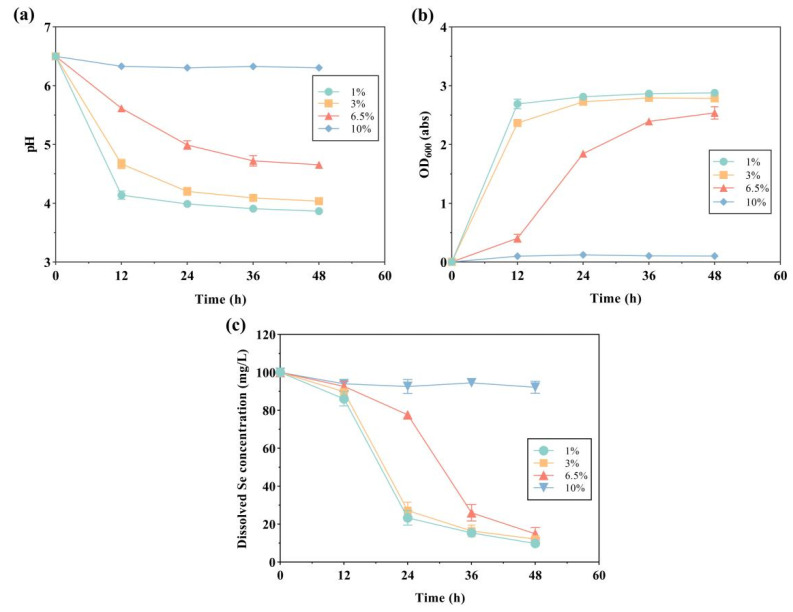
The effect of salinity on pH changes, OD_600_, and selenite reduction by strain LAB-Se2. Changes in (**a**) pH, (**b**) optical density, and (**c**) dissolved Se (IV) concentration. Error bars indicate the standard deviation (*n* = 3).

From the experimental results, it was observed that strain LAB-Se2 showed high salt tolerance (Figure 4). Strain LAB-Se2 was able to grow at a salinity up to 6.5% and still perform Se (IV) reduction. Sangmin et al. reported that the growth of strain NDSe-7 was not inhibited under the addition of selenium at 7% salinity [22]. Based on its salt tolerance, strain LAB-Se2 was considered to be effective for the treatment of selenium-containing wastewater environments [28]. No inhibition of growth or Se (IV) reduction was found in the salinity range of 1–3%, and Se (IV) clearance could reach about 90% after 48 h of incubation (Figure 4c). At a salinity of 6.5%, the growth of strain LAB-Se2 was slightly inhibited (Figure 4b). The optical density of the medium increased rapidly, and Se (IV) was rapidly reduced after 12 h of growth arrest. The clearance of Se (IV) at 48 h at a salinity of 6.5% was close to that at 3% salinity. When the salinity of the medium increased to 10%, the growth of strain LAB-Se2 was completely inhibited. The Se (IV) clearance rate of strain LAB-Se2 was basically the same in the salinity range of 1–6.5%, but it could be observed that high salinity (6.5%) had an inhibitory effect on the growth of bacteria through the measurement of the optical density of the medium.

### 2.4. Identification of Bacterial Strain LAB-Se2

**Figure 5:**
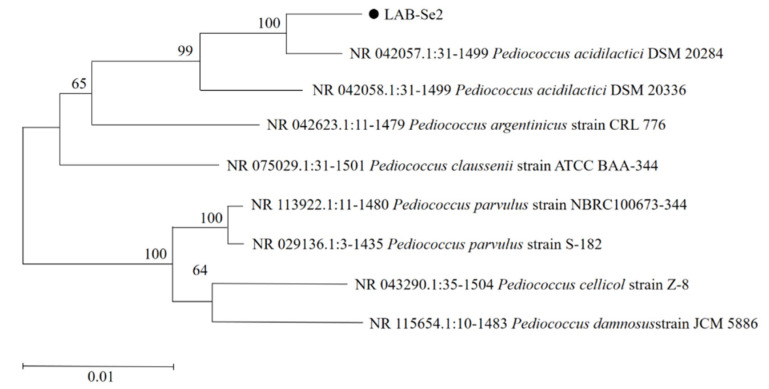
Phylogenetic tree of LAB-Se2 strain sequence alignment system.

A 1476 bp fragment of the 16S rRNA gene of the strain was obtained, and the strain LAB-Se2 was identified as *Pediococcus acidilactici* DSM 20284 (GenBank accession no. NR_042057) based on a literature survey review and BLAST results. The phylogenetic analyses derived from the neighbour-joining method showed the position of strain LAB-Se2’s position among species of the genus *Pediococcus* (Figure 5). Previous studies have shown that *P. acidilactici* exhibits suitable probiotic properties in vitro and can therefore be considered for development as a feed supplement [29].

According to recent research, the probiotic strain JZ07 of *Lactobacillus paralimentarius* accumulated Se from the tested medium at quite high levels [30]. The accumulated selenium was applied to the synthesis of selenium nanoparticles, and the excess selenium could be converted to elemental selenium, endowing the culture medium with a distinct red colour. In another study, *Lactobacillus paracasei* was found to accumulate Se by growing in medium enriched with 60 mg L^−1^ Se [25].

### 2.5. Biosynthesis of SeNPs

**Figure 6:**
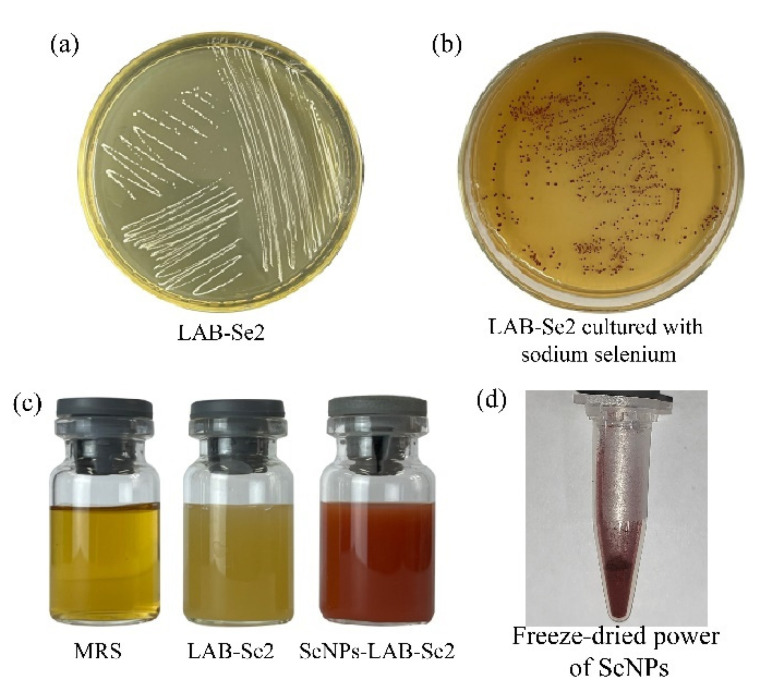
Preparation of SeNPs by LAB-Se2. Growth of strain LAB-Se2 on MRS agar plates without (**a**) and with (**b**) 100 mg L^−1^ selenite. (**c**), MRS; LAB-Se2, strain LAB-Se2 cultured in MRS; SeNPs-LAB-Se2, strain LAB-Se2 cultured in MRS with selenite. (**d**), Freeze-dried power of SeNPs. The red colony colour indicates that the strain reduced selenite to elemental selenium (Se^0^). Error bars indicate the standard deviation (*n* = 3).

During the whole culturing process, we observed that strain LAB-Se2 showed red colonies when cultured on MRS agar plates containing 100 mg L^−1^ sodium selenite (Figure 6b). The MRS culture medium without sodium selenite and the supernatant of the strain LAB-Se2 culture without sodium selenite were bright yellow after 48 h of incubation (Figure 6c). However, the culture broth of strain LAB-Se2 co-cultured with sodium selenite was dark red (Figure 6c). Selenium nanoparticles were extracted from strain LAB-Se2 after centrifugation. After freeze-drying, the SeNPs extracted from strain LAB-Se2 cells had a deep red colour (Figure 6d). It was generally considered that the zero-valent selenium in redox, identifiable by the grey and black colour of elemental selenium, belonged to the biologically inert form of selenium; however, a large number of studies confirm that nano-sized red elemental selenium synthesised by microorganisms has biological activity. This nanoform of selenium has the same biological activity as inorganic selenium and has the characteristics of low toxicity and easy absorption. In recent years, nano-selenium has attracted much attention because it was considered to be the safest form of selenium due to its low cytotoxicity and great biological activity [31].

### 2.6. Characterisation of SeNPs

#### 2.6.1. SeNPs Produced by Strain LAB-Se2 Contain an Organic Capping as Revealed by FTIR Spectroscopic Analysis

**Figure 7:**
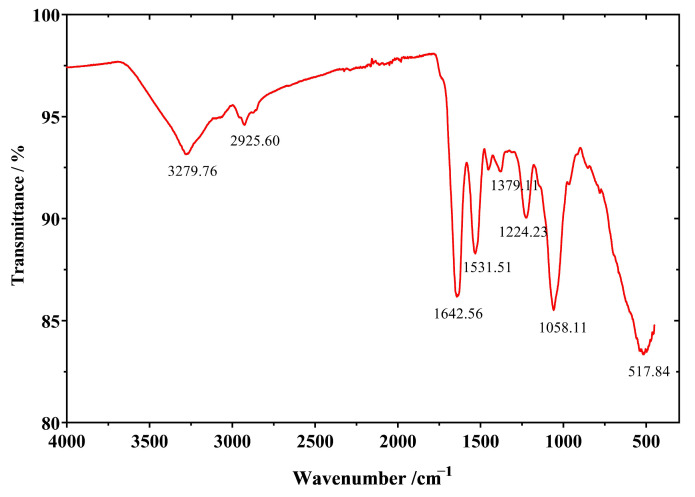
The FTIR spectrum of SeNPs synthesised by strain LAB-Se2.

The synthesis of SeNPs was confirmed, and the functional groups were observed by FTIR spectrometry. The functional groups involved in the reduction of sodium selenite to SeNPs were evaluated by the Fourier transform infrared spectroscopy of all SeNPs synthesised. The spectrogram (Figure 7) suggests that macromolecules such as lipids, sugars, carbohydrates, nucleic acids, and especially proteins may be present to ensure the stability of abiotic SeNPs [32]. The peaks were between 4000 and 500 cm^−1^. The strong absorption peak at 3286 cm^−1^ is due to combination of the stretching vibrations of the O–H and N–H bonds, which confirmed the presence of proteins and polysaccharides [9]. The broad band at 2925.60 cm^−1^ represents the stretching vibration of the C–H aliphatic bond of hydrocarbon functional groups. The sharp peak at 1642.56 cm^−1^ indicates the N–H bending of amide I or the C=O stretching of ester groups, while another peak at 1531.51 cm^−1^ is attributed to the C–H bending of alkene, representing the amide II band of protein [26]. The peak at 1379.11 cm^−1^ may be due to the O–H bending of carboxylate. The other band at 1224.23 cm^−1^ is attributed to amide III, while the band at 1064 cm^−1^ is assigned to the C–O stretching vibration of the polysaccharides. According to the FTIR analysis, protein capping and polysaccharides were confirmed to be present during the synthesis of SeNPs [10,12].

#### 2.6.2. Particle Size, Zeta Potential Measurements, and TEM Analysis

**Figure 8:**
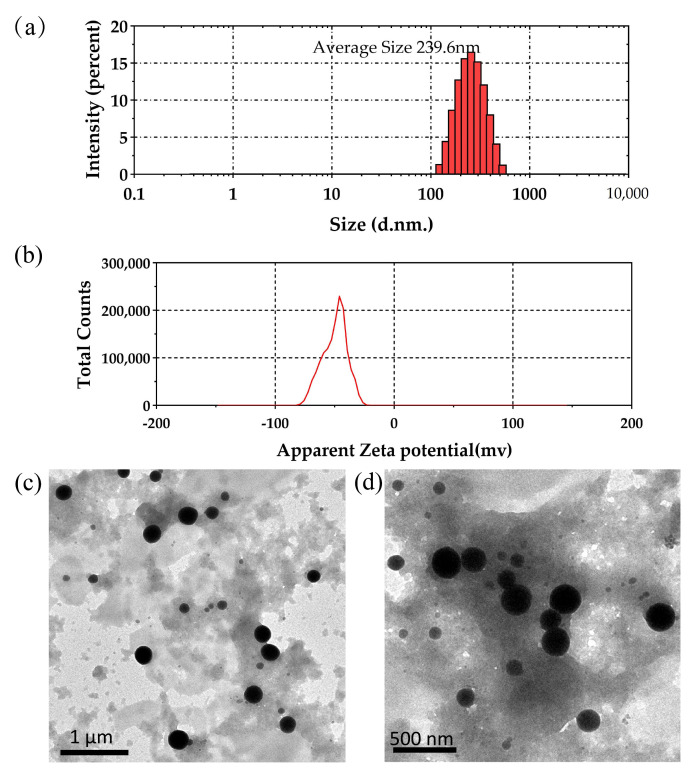
Particle size distribution (**a**), zeta potential (**b**), and TEM image (**c**,**d**) analyses of SeNPs biosynthesised by strain LAB-Se2.

The results of particle size and zeta potential analyses are shown in Figure 8. DLS analysis showed that the size of the purified SeNPs synthesised by strain LAB-Se2 was between 100 and 500 nm, the average particle size of the SeNPs was 239.6 nm (Figure 8a), and the PDI was 0.096, which indicated that the nanoparticles had great dispersion and were highly resistant to aggregation, which is similar to the size distribution trend found in other bacteria, such as *P. mirabilis* YC801 (178.3 ± 11.5 nm) [7] and *Bacillus subtilis* (217.11 nm) [21]. In the case of *Providencia* sp. *DCX* [20], selenium nanoparticles with an average size of 120 nm were generated under an argon atmosphere. In addition, in earlier studies carried out using haloalkaliphilic strain *B. Lysinibacillus*, selenium nanoparticles in the size range of 200–500 nm were synthesised [22]. Thus, our results on particle size were consistent with the biological production of selenium nanoparticles using different strains of bacteria. The difference in this study was that selenium nanoparticles were produced with an average particle size of 239 nm under both anaerobic and aerobic conditions.

The zeta potential is an important indicator of the colloidal dispersion stability of nanoparticles. Nanoparticles with a higher zeta potential exhibit greater stability due to greater electrostatic repulsion between nanoparticles [33]. In this study, the ZP of freeze-dried SeNPs synthesised by strain LAB-Se2 was −26.9 mV when suspended in deionised water (Figure 8b); thus, it was concluded that the SeNPs’ surfaces were negatively charged. Our results are consistent with the previous literature [34]. The morphology of biosynthesised SeNPs were assessed by TEM. Figure 8c,d shows the TEM images of the purified SeNPs at a resolution of 1 μm and 500 nm, respectively, where spherical Se nanoparticles could be seen. These particles varied in size (about 50–400 nm in diameter), similar to the size of nano-selenium produced by *Lysinibacillus bacilli* (50–400 nm in diameter) [22].

#### 2.6.3. Spectroscopic and Ultramicroscopic Analyses Revealed the Shape and Size of SeNPs

**Figure 9:**
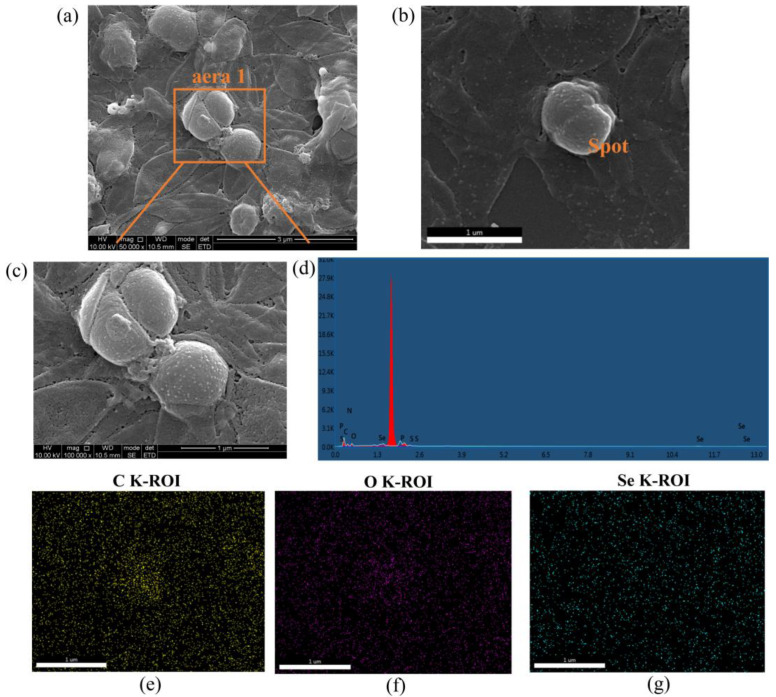
Characterisation of SeNPs synthesised by strain LAB-Se2 detected by SEM and EDS. (**a**) SEM image of the SeNPs secreted by strain LAB-Se2; (**b**) SEM image indicating the mapping region; (**c**) amplified image of area 1 in (**a**); (**d**) EDS spectrum of mapping region; (**e**) mapping for C element; (**f**) mapping for O element; and (**g**) mapping for Se element.

The morphology of SeNPs were analysed by SEM and EDS mapping (Figure 9). A representative SEM micrograph of the purified SeNPs is shown Figure 9a, where spherical and sphere-like selenium nanoparticles can be observed [7]. The elemental analysis of SeNPs (Figure 9b) was carried out by EDS-coupled SEM. As shown in Figure 9d, the EDS mapping showed a strong and sharp signal at 1.7 keV, which confirmed that elemental Se is present in the nano-selenium particles. Through EDS mapping, a strong peak at 1.37 keV proved the presence of elemental Se in the SeNPs produced by the two *Lysinibacillus* sp. strains [35]. Moreover, SEM and EDS images of the isolated SeNPs showed that carbon (C), oxygen (O), nitrogen (N), sulphur (S), and phosphorus (P) were also included in the elemental composition of the SeNPs [22], which was consistent with the detection of the presence of proteins in the FTIR analysis (Figure 7).

### 2.7. Bioactivities of SeNPs

#### 2.7.1. Antibacterial Activity

**Figure 10:**
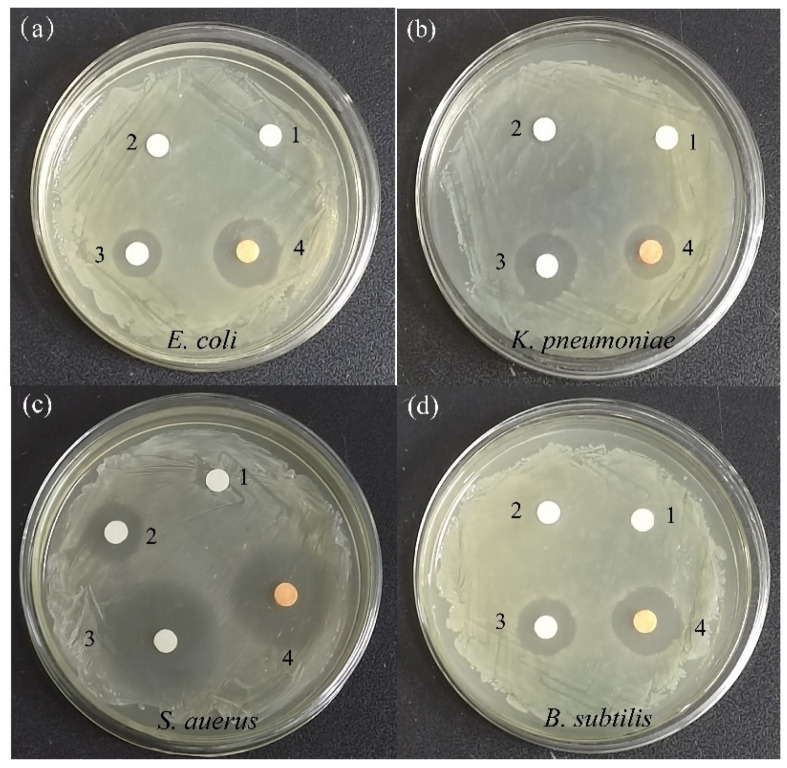
Antibacterial activity (zone of inhibition in mm) of SeNPs against (**a**) *E. coli*; (**b**) *K. penumoniae*; (**c**) *S. aureus*; (**d**) *B. subtilis* using disc diffusion method (1: deionised water, 2: sodium selenite, 3: ampicilin, 4: SeNPs).

**Figure 11:**
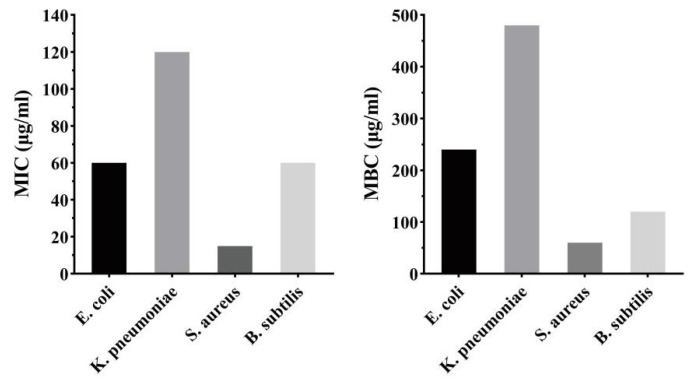
MICs and MBCs of SeNPs against Gram-positive and Gram-negative bacteria.

Recently, SeNPs have been considered as an antimicrobial agent against a variety of pathogenic microorganisms as a promising new strategy for the treatment of bacterial infections. In the present research, the antibacterial activity of the purified SeNPs was evaluated against Gram-positive bacteria (*S. auerus* and *B. subtilis*) and Gram-negative bacteria (*E. coli* and *K. pneumoniae*) through a disk diffusion assay (Figure 10). The appearance of an inhibition zone indicates that bacteria cannot grow, demonstrating the antibacterial effectiveness of a treatment. A filter disc soaked in deionised water had no inhibition effect, but sodium selenite showed different levels of inhibition toward indicator bacteria. The results showed that SeNPs cultured for 48 h showed substantial inhibition to the growth of *E. coli*, *K. pneumoniae*, *S. aureus*, and *B. subtilis* at a concentration of 100 mg L^−1^, with inhibition zones of 17.5 mm, 13.4 mm, 27.9 mm, and 16.2 mm, respectively (Table 1). In comparison, the broad-spectrum antibiotic ampicillin produced inhibition zones against these bacteria that were 14.1 mm, 16.0 mm, 30.2 mm, and 19.1 mm, respectively. The produced SeNPs had a remarkable antibacterial effect on *S. aureus* (30.2 ± 1.3 mm) compared with 30 µg mL^−1^ ampicillin. SeNPs had a slightly higher antimicrobial efficacy on *B. subtilis*, which was 19.1 ± 1.0 mm higher than the inhibition zone produced by ampicillin. MIC and MBC were also used to assess the antibacterial capacity of the synthesised SeNPs. In this study, the MICs of SeNPs for *E. coli*, *K. pneumoniae*, *S. aureus*, and *B. subtilis* were 60 µg mL^−1^ (*E. coli*), 120 µg mL^−1^ (*K. pneumoniae*), 15 µg mL^−1^ (*S. aureus*), and 60 µg mL^−1^ (*B. subtilis*), respectively. Among the four tested bacteria, SeNPs exhibited the lowest inhibitory concentration on *S. aureus* at 60 µg mL^−1^ (Figure 11). Meanwhile, SeNPs showed different MBCs for *B. subtilis*, *K. pneumoniae*, and *E. coli* of 120 µg mL^−1^, 480 µg mL^−1^, and 120 µg mL^−1^, respectively. These results indicate that the synthesised SeNPs were more effective against Gram-positive than Gram-negative bacteria. Similar results were reported in previous studies using biogenic SeNPs produced from bacteria [15,36], fungi [37], and plant extracts [34].

#### 2.7.2. Antioxidant Activity

According to previous reports, SeNPs have strong antioxidant activity, with significant scavenging effects on DPPH radicals, ABTS radicals, hydroxyl radicals, and superoxide radicals [3,13,15,34].

**Figure 12:**
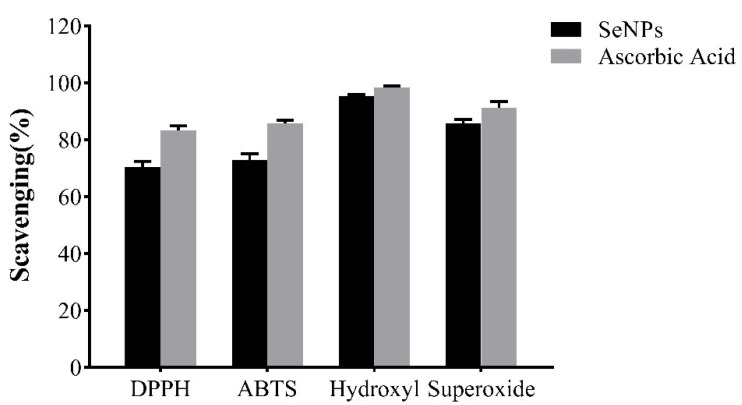
Antioxidant activity of SeNPs.

The different percent inhibitions of free radicals and ascorbic acid were compared to estimate the antioxidant activity of the preparation of SeNPs. The results showed that SeNPs were effective in scavenging four kinds of free radicals (Figure 12). The DPPH, ABTS, hydroxyl, and superoxide-scavenging activities of SeNPs were 70.3%, 72.8%, 95.2%, and 85.7%, respectively, whereas those of ascorbic acid were 83.4%, 85.7%, 98.2%, and 91.3%, respectively. Previous research showed that the DPPH and ABTS scavenging activities of SeNPs prepared by *Bacillus subtilis* BSN313 were 71.25% and 63.01%, which was consistent with the present study [15]. In the same way, EPS-BioSePs synthesised via *Bacillus paralicheniformis* SR14 exhibited higher antioxidant activities on DPPH, superoxide, and ABTS free radicals [38]. SeNPs showed a high hydroxyl-scavenging ability similar to ascorbic acid, which was contrary to the results of Cheng Yuanzhi [38]. These results indicate that SeNPs could possibly serve as potential antioxidants due to the functional groups that bind to them [39].

## 3. Material and Methods

### 3.1. Sample Collection and Strain Isolation

The soil sampling site is located in XinXiang (113° N, 35° E), Henan province of China, with a total Se content of 0.48 mg kg^−1^. These samples were collected from plant rhizo-sphere soils 10–30 cm below the surface in alfalfa fields. The whole soil samples were collected with sterile spoons and quickly placed into 45 mL sterile falcon tubes and transported to the lab on ice.

MRS medium was used to isolate selenite-reducing bacteria. The MRS medium was supplemented with a stock solution of Na_2_SeO_3_, with a final concentration of 100 mg L^−1^, to isolate the selenite.

In order to isolate selenite-reducing bacteria, the MRS medium was supplemented with a stock solution of Na_2_SeO_3_, with a final concentration of 100 mg L^−1^. After 2 days of incubation at 37 °C, colonies that had a reddish colour in the initial separation broth were re-incubated under the same conditions and kept for 48 h at room temperature. The agar plates without sodium selenite were regarded as controls. Colonies with a red colour, indicative of the reduction of selenite to elemental selenium, were streaked onto fresh plates to obtain isolates and kept in mixture of MRS medium and 30% glycerol at −80 °C for further experiments.

### 3.2. Selenite Reduction and Se Nanoparticle Production by Four Isolated Bacteria

Each bacterial isolate was grown in the same culture medium as used for its enrichment. In addition, the reduction of Se (IV) and the production of Se nanoparticles were detected via that media. Se (IV) was added to all culture media at a concentration of 100 mg L^−1^. After inoculation, the bacteria were incubated at 25 °C and 200 rpm for 3 days. A sample was taken from each culture every 12 h during the experiment to monitor the optical density of cell growth, pH value, and the concentration of Se (IV).

### 3.3. Selenite Reduction and Se Nanoparticle Production by Strain LAB-Se2 under Various Conditions

The strain with the highest Se (IV) removal rate was further studied, and the experimental parameters were optimised. In order to obtain the best conditions for selenite reduction and nanoparticle production, the growth of the LAB-Se2 strain was investigated under different initial pH levels, temperatures, and salinities (Table 2). To explore the effects of pH values, the pH of the medium was adjusted to 2.5–5.0 by adding HCl and NaOH solutions, keeping a constant salinity of 1.0% and a temperature of 35 °C. To research the effects of temperatures, the initial pH was maintained at 6.5, and the salinity was kept at 1.0%. They were cultured at 25, 30, 35, and 40 °C, respectively. To investigate the influence of salinity, the temperature was kept at 37 °C and the initial pH value at 6.5, while the salinity of medium was changed in the range of 1.0%–10% by adding a suitable amount of NaCl. Over an incubation period of 48 h, the optical density of cell growth, the pH value, and the concentration of Se (IV) were monitored every 12 h.

### 3.4. Phylogenetic Analysis of New Selenite-Reducing Bacteria

The genomic DNA was extracted from isolates by using the HiGene™ Genomic DNA Prep Kit according to the manufacturer’s protocol. The 16S rRNA gene was amplified using universal primers 27F (5′-AGAGTTTGATCMTGGCTCAG-3′) and 1492R (5′-TACGGYTACCTTGTTACGACTT-3′). The PCR products were sent to the Qingke Company (Beijing, China) to purify and sequence [9]. The obtained gene sequence was searched via the National Center for Biotechnology Information (NCBI)’s website. The phylogenetic sequences obtained from phylogenetic analysis and related sequences preserved in GenBank database were used to predict the evolutionary history of isolated strains. MEGA 7.0 software was used to construct the maximum likelihood tree based on the neighbour-joining method, and 1000 replicates were bootstrapped [8]. The GenBank database was given an accession number NR_042057 for the sequence that had been generated.

### 3.5. Determination of Selenite Content

The ability of Lactobacilli to reduce selenite was determined by culturing strains in MRS supplemented with 100 mg L^−1^ Na_2_SeO_3_ at 37 °C for 48 h. The grown cultures were centrifuged at 7500× *g* for 10 min, and the remaining Na_2_SeO_3_ concentration in the supernatant was determined using a modified version of the microplate assay spectrophotometry method by Brown and Watkinson [40]. Briefly, 1 mL supernatant was mixed with 34 mL purified H_2_O and 1 mL 5% EDTA-2Na in a 100 mL beaker, and the pH was adjusted to 2–3 with 0.5 M HCl. Next, 4 mL of 0.5% DAB was added, the solution was shaken well, and the pH was adjusted to neutral. The liquid was transferred from the beaker to a separate funnel, and 10 mL of toluene was added. The mixture was shaken vigorously for 3 min to fully extract inorganic selenium with the toluene and then left to stratify in the dark for 30 min. The water layer was discarded, while the toluene layer was filtered and collected. The absorbance of the toluene layer at the wavelength of 420 nm was measured by a UV spectrophotometer. All measurements were performed in triplicate.

### 3.6. Biogenic Synthesis of Selenium Nanoparticles by Strain LAB-Se2

For the biosynthesis of SeNPs, 2% (*v*/*v*) of strain LAB-Se2 was added to 100 mL of sterilised MRS medium with 100 mg L^−1^ Na_2_SeO_3_. After 48 h of aerobic cultivation at 37 °C in a shaking incubator, the bacterial culture with a red colour was centrifuged for 10 min at 13,000× *g* and then washed three times with sterile water. Lysozyme (20 mg mL^−1^) was added to the thallus precipitation and incubated at 37 °C for 20 min. During this period, the container was taken out and turned upside down several times every 5 min to ensure the solution was fully mixed. Then, the cell walls were broken by ultrasound for 30 min (4 °C) under high pressure (300 W) at 2 s intervals, and the intracellular nanoparticles were released into the aqueous solution. The supernatant was recovered after centrifuging for 10 min at 6000× *g*; following that, the supernatant was centrifuged at 13,000× *g* for 10 min and then discarded, and the precipitate was washed out with deionised water and stored. For further characterisation, the purified SeNPs were freeze-dried at 0.12 mbr and −40 °C for 24 h [15].

### 3.7. Characterisation of SeNPs 

The biosynthesised selenium nanoparticles were characterised to determine their sizes, shape, and surface area. The techniques used to characterise SeNPs were transmission electron microscopy (TEM), scanning electron microscopy (SEM) assisted with energy dispersive spectrometry analysis (EDS), and Fourier transform infrared (FTIR) spectroscopy.

#### 3.7.1. FTIR Spectroscopic Analysis

An infrared Fourier transform spectrometer (FT-IR, PerkinElmer, Spectrum GX, Waltham, MA, USA) was used to examine the functional groups within the SeNPs responsible for reducing and capping the nanoparticles by scanning over the frequency range of 4000–400 cm^−1^ [8].

#### 3.7.2. Particle Size and Zeta Potential Measurements

Synthetic SeNPs were characterised using a dynamic light scattering (DLS) particle size analyser (ZerasizerNano ZS, Malvern, UK) to determine the particle size, polydispersity index (PDI), zeta potential value, and particle size distribution (PSD) [24]. SeNPs were dissolved in deionised water and mixed thoroughly for 20 min before transferring about 0.5 mL of the suspension to the cuvette of the dip cell kit for ZP and EM measurements to determine particle size.

#### 3.7.3. Transmission Electron Microscopy

To determine the morphology and size of selenium nanoparticles, a purified sample suspended in deionised water was drop-coated on a copper grid and air-dried. Subsequently, the SeNPs were observed using TEM (JEOL JEM-1010) operating at a 100 kV accelerating voltage. Finally, images were captured and analysed using an analysis software.

#### 3.7.4. Scanning Electron Microscopy with EDS

The shapes and elements of the SeNPs were determined using scanning electron microscopy (JSM-IT 100, JEOL, Tokyo, Japan) coupled with EDS. Selenium nanoparticles suspended in water were carefully drop-coated onto a carbon stub using sticky tape and evenly coated with carbon (JEOL-EC-32010CC). Scanning of SeNPs was performed at 10 kV with various magnifications varying from ×10,000 to ×100,000. To prove the existence of elemental selenium, an EDS examination was conducted [22].

### 3.8. Antimicrobial Activity of SeNPs

#### 3.8.1. Disk Diffusion Method

Through the agar disk diffusion method, the antimicrobial activity of SeNPs against *E. coli*, *B. subtilis*, *S. aureus*, and *K. pneumoniae* was determined. Isolates cultured overnight were inoculated on LB plates. SeNPs prepared with 100 mg L^−1^ sodium selenite were used for antibacterial experiments. A concentration of antibiotics (ampicillin) was used as positive controls, and blank disks treated with sterile water were used as negative controls. After incubating at 37 °C for 48 h, the inhibition zone was determined in mm with a ruler.

#### 3.8.2. Minimal Inhibitory Concentration (MIC) Test

To determine minimal inhibitory concentration (MIC) of SeNPs, synthesised SeNPs were prepared in different concentrations ranging from 480 to 7.5 μg mL^−1^, which were then assessed separately to detect MIC against selected bacterial strains [41]. MICs were defined as the lowest concentrations that completely inhibited the growth of bacteria when viewed with an unaided eye [42]. All determinations were performed in triplicate.

#### 3.8.3. Minimum Bactericidal Concentration (MBC) Test

After the determining the MIC of the SeNPs, aliquots of 100 μL from all tubes which showed no visible bacterial growth were seeded in nutrient agar plates. Any organisms that were inhibited but not killed in the MIC test now had a chance to grow because the SeNPs had been diluted significantly. After a standard incubation, the lowest concentration of antimicrobial agent that reduced the number of colonies by 99.9% is defined as the MBC [43]. All determinations were performed in triplicate.

### 3.9. Antioxidant Activity of SeNPs

#### 3.9.1. DPPH Scavenging Assay

Synthesised SeNPs were evaluated for their antioxidant activity using DPPH radical scavenging [44]. The DPPH solution was formulated in a ratio of 1 mL of DPPH to 20 mL of methanol. A certain amount of SeNPs and DPPH solution were thoroughly mixed, and then cultured in the dark for 30 min. Afterwards, a UV-Visible spectrophotometer was used to estimate the absorbance at 517 nm. Ascorbic acid was used as a control. The DPPH-radical-scavenging activity was calculated as follows:

$$DPPH\;radical\;scavenging\;activity\;(\%)=\frac{A_{a}-A_{b}+A_{c}}{A_{a}}\times 100\%$$

where *A_a_* is the absorbance of the DPPH solution without the sample as a blank control, *A_b_* is the absorbance in the presence of the tested sample, and *A_c_* is the absorbance of the sample.

#### 3.9.2. ABTS Scavenging Assay

The method of determining SeNPs’ ABTS-radical-scavenging activity was slightly modified [45]. The reaction between 7.4 mM ABTS and 2.5 mM potassium persulfate was carried out in water for 12 h at a ratio of 1:1 to obtain an ABTS radical solution. Then, the ABTS radical solution was diluted with methanol, and the optical density was adjusted to 0.7 at 734 nm. After that, 1 mL of SeNPs was added to 3 mL of ABTS solution (optical density of 0.7) and cultured for 6 min in the dark. The optical density (OD value) at 734 nm was measured with a UV-Visible spectrophotometer. Ascorbic acid was used as a control. The ABTS-radical-scavenging activity was calculated as follows:

$$ABTS\;radical\;scavenging\;activity\;(\%)=\frac{A_{a}-A_{b}+A_{c}}{A_{a}}\times 100\%$$

where *A_a_* is the absorbance of the DPPH solution without the sample as a blank control, *A_b_* is the absorbance in the presence of the tested sample, and *A_c_* is the absorbance of the sample.

#### 3.9.3. Hydroxyl Scavenging Assay

According to the method in the research report by Smirnoff and Cumbes [46], the scavenging activity of selenium nanoparticles against hydroxyl radicals was determined. First, 1 mL SeNPs solution was added to a reaction mixture containing 1 mL iron sulphate (1.5 × 10^−3^ mol L^−1^), 0.7 mL hydrogen peroxide (6 × 10^−3^ mol L^−1^), and 0.3 mL sodium salicylate (2 × 10^−2^ mol L^−1^). The mixture was then incubated at 37 °C for 60 min, and the absorbance was measured at 562 nm. The absorbance of the reaction solution was then measured at 562 nm after incubation for 60 min at 37 °C. Ascorbic acid was used as a control. The hydroxyl-radical-scavenging activity was calculated as follows:

$$Hydroxyl\;radical\;scavenging\;activity\;(\%)=\frac{A_{a}-A_{b}+A_{c}}{A_{a}}\times 100\%$$

where *A_a_* is the absorbance of the DPPH solution without the sample as a blank control, *A_b_* is the absorbance in the presence of the tested sample, and *A_c_* is the absorbance of the sample.

#### 3.9.4. Superoxide Scavenging Assay

The method of determination of the SeNPs’ superoxide-radical-scavenging activity was slightly modified [47]. Briefly, a sample solution of 100 μL gradient concentrations (0.1, 0.2, 0.25, 0.5, 1, and 2 mM) was mixed with 100 μL or 150 μM NBT solution and 100 μL or 470 μM β-NADH solution and then thoroughly mixed. After intensive mixing, 20 μL or 60 μM PMS solution was added and then left to set at room temperature for 5 min. As the control group, 100 μL of the sample solution was mixed with 220 μL double-distilled water. Absorbance was measured at 560 nm. Ascorbic acid was used as a control. The superoxide-radical-scavenging activity was calculated as follows:

$$Superoxide\;radical\;scavenging\;activity\;(\%)=\frac{A_{a}-A_{b}+A_{c}}{A_{a}}\times 100\%$$

where *A_a_* is the absorbance of the DPPH solution without the sample as a blank control, *A_b_* is the absorbance in the presence of the tested sample, and *A_c_* is the absorbance of the sample.

### 3.10. Statistical Analysis

The graphs in this study were all drawn with GraphPad Prism Version 8.0 and Origin Version 2018.

## 4. Conclusions

It is concluded from our results that the green synthesis of selenium nanoparticles was carried out successfully by using the strain *P. lactis*, which could remove 98% of selenium within 48 h at a 100 mg L^−1^ sodium selenite concentration and produced spherically shaped SeNPs with a size of about 239 nm. The green-synthesised SeNPs showed enhanced antibacterial activity against Gram-positive bacteria (*S. auerus* and *B. subtilis*) and exhibited optimal MIC values against *S. auerus* (15 μg mL^−1^). Importantly, SeNPs showed promising antioxidant activity on DPPH, ABTS, hydroxyl, and superoxide radicals. The present study shows that *P. lactis*-based SeNPs could have significant potential to be further developed for use in such applications as diet additives and in biomedicine; however, further studies are necessary to clarify the mechanisms involved.

## Figures and Tables

**Table 1:** Antibacterial effects of SeNPs produced by DSM20284.

Organisms	Diameter of Zone of Inhibition (in mm)			
	Deionised Water	Sodium Selenium	Ampicillin (10 μg/disk)	SeNPs
*E. Coli*	0.0	8.0 ± 1.1	14.1 ± 1.0	17.5 ± 0.8
*K. pneumoniae*	0.0	8.6 ± 1.0	16.0 ± 1.1	13.4 ± 0.9
*S. aureus*	0.0	13.1 ± 0.9	30.2 ± 1.3	27.9 ± 1.2
*B. subtilis*	0.0	7.6 ± 1.1	19.1 ± 1.0	16.2 ± 1.1

**Table 2:** Experimental conditions for the optimisation of Se (IV) reduction by strain LABSe-2.

Environmental Factors	Initial pH	Temperature/°C	Salinity/%
Initial pH	2.5	35	1.0
	3.0		
	3.5		
	4.0		
	4.5		
	5.0		
Temperature	6.0	25	
		30	
		35	
		40	
Salinity	6.0	35	1.0
			3.0
			6.5
			10

## Data Availability

The data is available on request.

## Supplementary Materials

The following supporting information can be downloaded at: https://www.mdpi.com/article/10.3390/molecules28093793/s1, Figure S1: Isolated strains showing colonies on agar plates (without and with Na_2_SeO_3_). Concentration of Se (IV) in agar plates was 100 mg L^−1^; Figure S2: Bacterial cultures with 100 mg L^−1^ of Se (IV) (right flasks), together with abiotic controls (left flasks) and biotic controls without Se (IV) (middle flasks). (a) Strain LAB-Se2, (b) strain LAB-Se4, (c) strain LAB-Se5, (d) Strain LAB-Se7.

## Sample Availability

Samples of the compounds are available from the authors.
