# Supplementary material for: Biological Selenite Reduction, Characterization and Bioactivities of Selenium Nanoparticles Biosynthesised by Pediococcus acidilactici DSM20284
Source: Molecules. 2023 Apr 28;28(9):3793. doi: 10.3390/molecules28093793 (PMC10180234; doi:10.3390/molecules28093793)
Supplement: Supplementary file 1 [file molecules-28-03793-s001.zip › molecules-2336047-supplementary.pdf]

Supplementary Materials

Biological Selenite Reduction, Characterization and

Bioactivities of Selenium Nanoparticles Biosynthesised by *Pediococcus acidilactici*

DSM20284

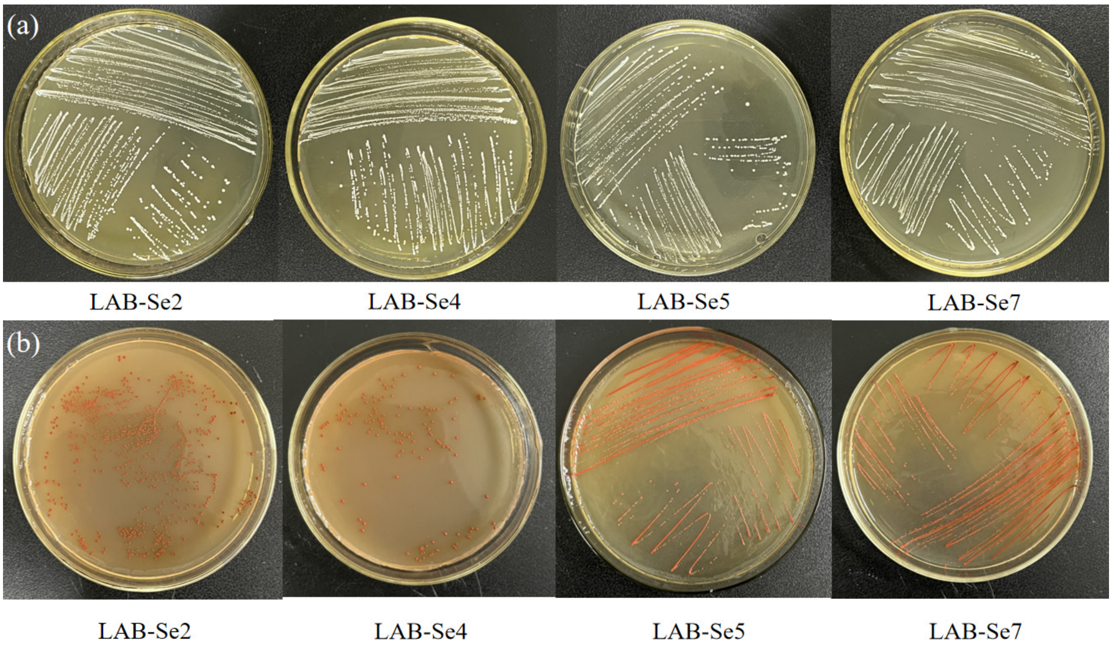

Figure S1. Isolated strains showing colonies on agar plates (without and with Na_2SeO_3). Concentration of Se (IV) in agar plates was 100 mg L^{-1}.

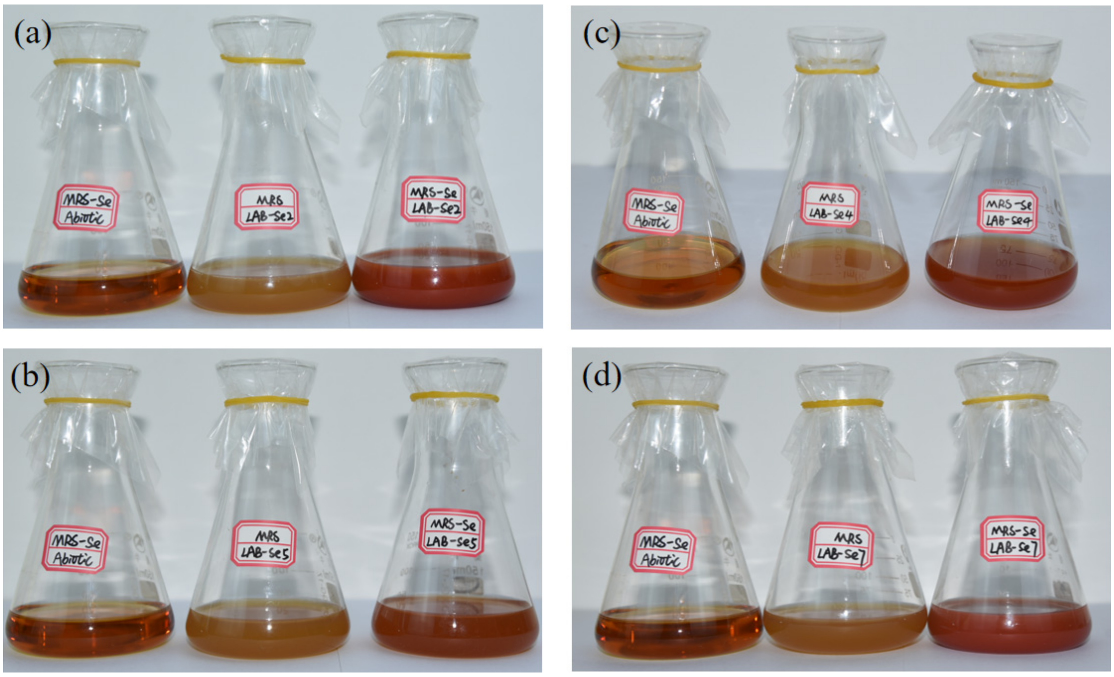

Figure S2. Bacterial cultures with 100 mg/L of Se (IV) (right flasks), together with abiotic controls (left flasks) and biotic controls without Se (IV) (middle flasks). (a) strain LAB-Se2, (b) strain LAB-Se4, (c) strain LAB-Se5, (d) Strain LAB-Se7.
